# Best Practices for Optimizing Patients Undergoing Surgical Procedures to Prevent Postoperative Venous Thromboembolism: A Quality Improvement Project

**DOI:** 10.7759/cureus.65929

**Published:** 2024-08-01

**Authors:** Cameron Gerhold, Liam T McLoughlin, Brady Culpepper, Renish N Contractor, Terrence Regan

**Affiliations:** 1 Orthopedic Surgery, Florida State University College of Medicine, Tallahassee, USA; 2 Obstetrics and Gynecology, Florida State University College of Medicine, Tallahassee, USA; 3 Family Medicine, Florida State University College of Medicine, Tallahassee, USA; 4 Urology, Florida State University College of Medicine, Tallahassee, USA; 5 Urology, AdventHealth Florida, Daytona Beach, USA

**Keywords:** pulmonary embolism (pe), deep vein thrombosis (dvt), venous thromboembolism (vte), caprini risk assessment, quality improvement and patient safety

## Abstract

Introduction

Current studies suggest that both chemical and mechanical venous thromboembolism (VTE) prophylaxis is underused, which is concerning due to the potential lethality of VTEs. The Caprini risk score is a preoperative VTE risk assessment that determines a patient's risk of enduring a VTE. The objective of this study was to examine postoperative cases of VTE to determine if accurate VTE risk stratification was performed and whether appropriate VTE prophylaxis was administered.

Methods

A retrospective analysis was conducted on 23 reported cases of VTE that occurred at a Central Florida hospital from April 1, 2021, to March 31, 2022. Relevant demographic and medical information was gathered from each patient chart to calculate an individual Caprini risk score and determine the type of chemical VTE prophylaxis that was received.

Results

Out of 23 reported cases of VTE in surgical patients, 17 were ultimately determined to have suffered VTE associated with their hospitalization and surgery. Thirteen out of 17 (76%) received appropriate perioperative chemical deep vein thrombosis (DVT) prophylaxis based on the calculated Caprini risk score and corresponding recommendations. Four out of 17 (24%) were determined to have received insufficient perioperative chemical DVT prophylaxis.

Conclusion

Consistent utilization of a DVT/pulmonary embolism (PE) risk stratification tool, such as the Caprini risk score calculator, is essential in the prevention of postoperative VTE. Hospitals can improve the utilization of such a tool and thereby reduce the number of embolic events by making it more visible and accessible to the overseeing provider in the electronic medical record (EMR).

## Introduction

Venous thromboembolism (VTE) is a serious and potentially life-threatening condition that can occur in surgical patients. VTE includes deep vein thrombosis (DVT) and pulmonary embolism (PE). Many organizations and societies have developed guidelines and recommendations for VTE prophylaxis in surgical patients. These guidelines include risk assessment tools to identify patients at increased risk for VTE, as well as recommendations for appropriate prophylaxis based on a patient's individual risk factors. Appropriate prophylactic measures may include pharmacological interventions, such as anticoagulants, and mechanical interventions, such as sequential compression devices. These measures have been shown to significantly reduce the incidence of VTE in surgical patients [1].

However, despite the availability of these guidelines and recommendations, VTE still occurs in some surgical patients. This may be due to a variety of factors, including patient-specific factors, failure to appropriately assess and identify patients at risk for VTE, or failure to implement appropriate prophylactic measures [2]. Therefore, while VTE is a preventable occurrence in surgical patients, it requires a multifaceted approach that includes appropriate risk assessment, implementation of evidence-based prophylactic measures, and ongoing monitoring and evaluation of outcomes to ensure that prevention efforts are effective [2].

In general, evidence-based guidelines recommend that patients undergoing major surgery should have mechanical prophylaxis and use pneumatic compression [3]. A review published in 2020, which looked at articles discussing VTE prevention in surgical patients, found that in non-orthopedic surgery, the overall approach to methods of prophylaxis involved risk assessment tools such as the Caprini score [4]. This is a scoring tool that includes most of the risk factors known to be associated with VTEs. It assigns point values to each risk factor to ultimately calculate an overall score of low, moderate, or high risk [5]. This is an important part of VTE prophylaxis done preoperatively, which helps physicians administer the proper chemical thromboprophylaxis.

Audits listed within a study conducted by Kahn et al. have suggested that chemoprophylaxis is underused [6,7]. Explanations for this have been identified by a 2022 review, which found consistently low rates of accurate risk stratification and appropriate chemoprophylaxis being applied in clinical practice [8]. Finally, a 2021 retrospective review showed that a significant increase in VTE prophylaxis administration occurred when a multifaceted approach was used. The key components of this approach included having a responsible provider who would be dedicated to VTE prophylaxis and a system in place to regularly check compliance [9].

As seen from the preceding literature, there are several challenges that face VTE prophylaxis in surgical patients. It can be difficult to balance the risk of VTE with the risk of bleeding. Compliance with the protocols that are set is a challenge as well; the physicians and nurses need to communicate well and have systems in place to monitor compliance. Validated risk assessment tools need to be used appropriately to best identify patients at increased risk for VTE.

The National Surgical Quality Improvement Program (NSQIP) is a program developed by the American College of Surgeons (ACS) that collects and analyzes data on surgical outcomes from participating hospitals. The program was established in 2001 with the goal of improving surgical quality by measuring and tracking surgical outcomes and using that data to inform quality improvement efforts. The NSQIP collects data on a wide range of variables, including patient demographics, preoperative risk factors, intraoperative variables, and postoperative outcomes. These data are used to develop risk-adjusted models that can help hospitals compare their surgical outcomes to national benchmarks and identify areas for improvement. The NSQIP has specifically developed guidelines for VTE prophylaxis in surgical patients, and implementation of ACS NSQIP guidelines is associated with improved outcomes. It can be presumed, therefore, that greater efforts devoted toward the utilization of NSQIP data to direct quality improvement may lead to better surgical outcomes [10].

This study aims to investigate instances of VTE that occurred at a Central Florida hospital from April 1, 2021, to March 31, 2022, for evidence of improper VTE risk stratification or failure to provide appropriate chemical VTE prophylaxis.

## Materials and methods

After obtaining approval from the appropriate Institutional Review Board, we accessed the medical records of patients who experienced a postoperative VTE between April 1, 2021, and March 31, 2022. The Central Florida hospital surveyed in this study participates in NSQIP. We were provided access to their Surgical Health Equity data to view demographic and medical data needed to calculate a Caprini risk score. The following demographic and medical information were collected: (1) date of birth; (2) age at time of surgery; (3) sex; (4) BMI > 25; (5) type of surgery; (6) past major surgery (<45 min) within the past month; (7) visible varicose veins; (8) history of inflammatory bowel disease (IBD); (9) swollen legs; (10) myocardial infarction (MI) within past month; (11) congestive heart failure (CHF) within the past month; (12) serious infection (pneumonia, sepsis) within the past month; (13) existing lung disease (COPD or emphysema); (14) stroke within the past month; (15) bed rest or restricted mobility within the past month; (16) non-removable plaster cast; (17) removable leg brace or mold that has kept patient from moving their leg within the past month; (18) current or past malignancy; (19) central venous access port within the past month; (20) current or past history of DVT or PE; (21) family history of DVT or PE; (22) personal or family history of positive blood test indicating an increased risk of blood clotting; (23) fracture of the hip, leg, or pelvis; (24) multiple traumatic injuries; (25) spinal cord injury resulting in paralysis; (26) and administration of VTE prophylaxis.

**Figure 1:**
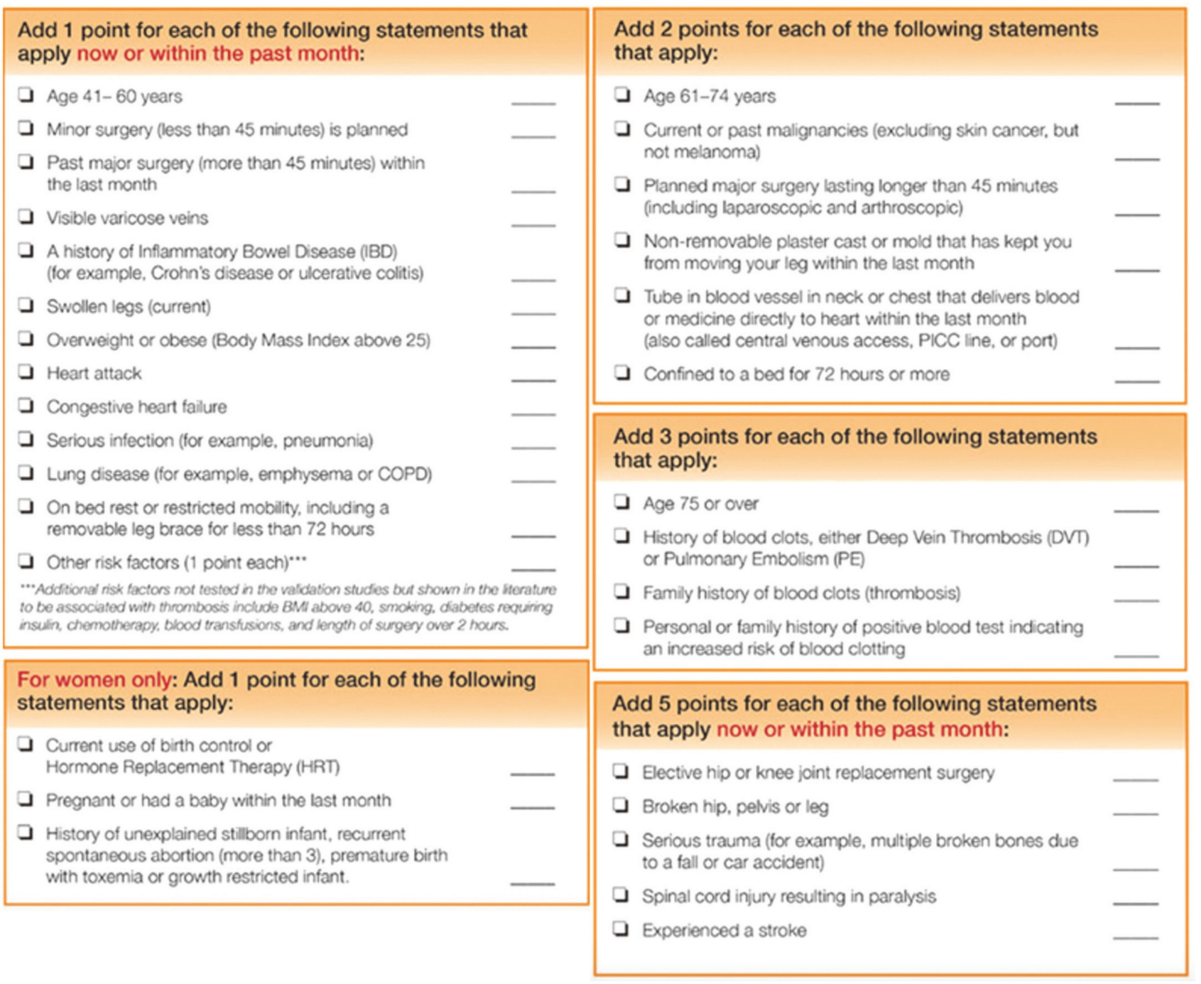
The 2013 Caprini risk score variables and points associated with each variable. Reference: [11]

The Caprini risk score (Figure 1) evaluates many factors, some of which contribute a greater role to the development of a VTE. Variables that play a more important part in the development of a VTE correspond with a higher number of points. A score of zero to four corresponds with a low VTE risk, a score of five to eight corresponds to a moderate VTE risk, and a score of nine or more corresponds with a high VTE risk.

After calculating a Caprini risk score, a search of the patient's medical record limited to the time during which VTE occurred was conducted to determine whether the patient received appropriate VTE chemoprophylaxis. The Caprini risk score calculator includes VTE prophylaxis recommendations based on the risk score calculated. These recommendations were used to determine whether patients received appropriate prophylaxis.

The retrospectively calculated Caprini risk scores were to be compared to the original Caprini risk scores calculated at the time of hospitalization, if available in the chart. Discrepancies between the scores were to be documented. We also aimed to determine whether patients were given the appropriate VTE chemoprophylaxis based on the originally calculated Caprini risk score, whether accurate or not. The overall accessibility of the Caprini risk score tool and visibility of the Caprini risk score from the perspective of a provider navigating the patient chart were noted while performing chart reviews.

Protection of confidential information

Practices for accessing and storing information from medical records were in accordance with the Health Insurance Portability and Accountability Act (HIPAA) guidelines. The use of procedural codes minimizes the likelihood of the study team accessing patient records not relevant to the study. The identifiable health information was stored in a secure password-protected folder that could only be accessed by members of the study team. Data were not banked for future studies. Only de-identified data may be released outside of the research team and the hospital's secure servers. The study team was trained on the hospital's electronic medical record (EMR), chart review protocols, and data storage protocols.

## Results

Seventeen out of 23 reported cases of VTE in surgical patients were ultimately included in the final analysis. Patients had an average age of 69 years. Eight out of 17 (47%) were female. Seven out of 17 (41%) underwent orthopedic procedures, while the remaining underwent general surgical procedures. Frequent comorbidities and risk factors included obesity, diabetes, CHF, lung disease, ongoing malignancy, a personal history of DVT or PE, and a long bone fracture. Patients had an average Caprini risk score of 9.9, corresponding to the highest category of VTE risk stratification. No disparities based on age, sex, or race were found. A full summary of descriptive statistics can be found in Table 1.

**Table 1:** Demographics and descriptive statistics. BMI: body mass index; kg: kilogram; m: meter; COPD: chronic obstructive pulmonary disease; CHF: congestive heart failure; HTN: hypertension; DVT: deep vein thrombosis; PE: pulmonary embolism

General Information	Number of Patients
Total patients	17
Sex	
Male	9
Female	8
Race	
Caucasian	15
African American	2
Hispanic	1
Average age (years)	69
Average BMI (kg/m^2^)	28.8
Average length of stay (days)	9.6 ± 9
Average length of surgery (minutes)	132 ± 89
Surgical Information	
Orthopedic procedures	7
General surgery procedures	10
General anesthesia	17
Insurance	
Patients with Medicare	14
Patients with private insurance	2
Self-pay patients	1
Comorbidities	
Diabetes	5
COPD	6
CHF	3
HTN with medication	14
Obesity	8
History of DVT or PE	4
Ongoing or history of malignancy	6
History of DVT or PE while on warfarin	1
Current smokers within the past year	4

**Figure 2:**
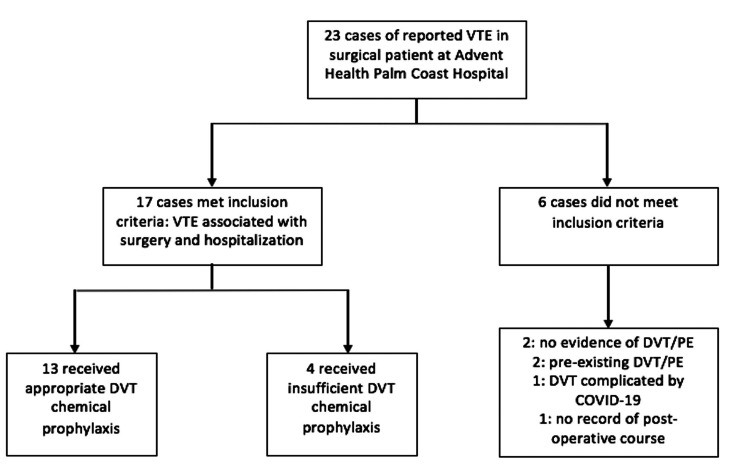
Flow chart of patient inclusion and exclusion based on the study criteria and prophylaxis administration. VTE: venous thromboembolism; DVT: deep vein thrombosis; PE: pulmonary embolism

Out of the 23 reported cases of VTE in surgical patients, 17 were ultimately determined to have suffered VTE associated with their hospitalization and surgery. The remaining six cases did not meet the criteria for inclusion. Of these, two patients showed no evidence of VTE in the EMR, two presented with the DVT of concern already present, and one suffered VTE complicated by COVID-19 infection several months following surgery. The remaining patient’s EMR contained insufficient information on the surgery and postoperative course (Figure 2).

Out of the 17 cases that met the inclusion criteria for VTE, 13 (76%) received appropriate perioperative chemical DVT prophylaxis based on the calculated Caprini risk score and corresponding recommendations. In these cases, patients initiated Eliquis, Xarelto, or heparin during the perioperative period or resumed chronic anticoagulation therapy promptly following surgery.

Four out of 17 (24%) were determined to have received insufficient perioperative chemical DVT prophylaxis. Two out of four patients underwent orthopedic procedures, which included shoulder arthroscopy and pin placement for fracture prophylaxis in a patient with metastatic cancer. These patients had an average Caprini risk score of 9, corresponding to a risk stratification level of “highest.” Neither patient showed evidence of receiving VTE prophylaxis during hospitalization or being discharged on aspirin or an alternative direct oral anticoagulant (DOAC). In both cases, the patient presented to the emergency department with DVT within three weeks of the initial surgery. The remaining two patients underwent open umbilical hernia repairs. The patient with a Caprini risk score of 5, corresponding to a risk stratification of “high,” showed no evidence of receiving VTE prophylaxis during hospitalization or being discharged on a DOAC. The patient with a Caprini risk score of 9 had been off chronic warfarin therapy for more than 10 days during the time of surgery. The patient had a history of DVT and PE while on warfarin. There was no evidence of either VTE prophylaxis administration during hospitalization or discharge on a DOAC. Both patients presented to the emergency department with lower extremity DVT within four weeks of initial surgery.

Despite hospital protocol that requests the calculation of a Caprini risk score for each patient undergoing surgery, we were unable to find a record of the original Caprini risk score calculated at the time of hospitalization in the patient's EMR. It is unknown whether scores are simply not visible in the EMR or if they were never calculated to begin with. However, all Caprini risk scores were calculated retrospectively.

## Discussion

Numerous risk factors and comorbid conditions have been described in the medical literature to increase the risk of venothrombotic events in surgical patients. Some significant risk factors for thrombosis in general surgery patients include existing metastatic disease, duration of procedure, previous VTE, advanced age, and obesity. Other medical conditions known to increase the risk of VTE include diabetes, COPD, heart disease, hypertension, bleeding disorders, and sepsis [12]. Our data support the current literature, as many of the patients who experienced a VTE had one or more of these comorbid conditions.

Consistent utilization of a DVT/PE risk stratification tool, such as the Caprini risk score calculator, is essential in the prevention of postoperative VTE. Numerous studies and audits have suggested that chemoprophylaxis is underused [7,13-17]. Explanations for this have been identified by a 2022 review, which found consistently low rates of accurate risk stratification and appropriate chemoprophylaxis being applied in clinical practice [8]. Currently, the Caprini risk score is not prominently featured on the patient's EMR home screen, nor is there a prompt to indicate the patient is not receiving appropriate prophylaxis. Hospitals such as Advent Health Palm Coast could improve the utilization of such a tool by making it more visible and accessible to the overseeing provider in the EMR. It may be beneficial to implement a feature in the EMR system that reminds the overseeing surgeon to complete a Caprini risk assessment. This assessment should then be made visible in the EMR to all healthcare staff attending to the patient.

In addition to the interventions previously described, the previous study team proposed implementing a new interactive VTE risk assessment tool, which would include a tip sheet or visual aid and a computer-based resource. The new risk assessment tool would be more interactive, allowing healthcare providers to ask questions and receive immediate feedback on the appropriate prophylactic measures for individual patients. Currently, this Central Florida hospital is implementing the Johns Hopkins everyBODYmoves initiative, a campaign that aims to address immobility harm in the acute hospital and post-acute setting. A future project may examine whether high-risk patients are receiving appropriate outpatient anticoagulation.

A limitation of this study is that we only accessed data from one healthcare site located within a suburban area of Florida. This greatly reduced our cohort size, and future studies should aim to gather data from multiple sites to gain a better understanding of whether VTE prophylaxis is utilized when recommended. Additionally, individuals under the age of 18 who sustained a DVT or PE were not included, as the focus of this study was adult patients. However, excluding this demographic may have limited our cohort size. Lastly, we only assessed patients who were diagnosed with a DVT or PE. Therefore, there is a likelihood that individuals who were recommended to be given VTE prophylaxis were administered the appropriate treatment. Thus, those patients did not suffer from a DVT or PE. There is an opportunity for studies to evaluate what proportion of total patients recommended to receive VTE prophylaxis end up being administered prophylaxis, regardless of whether they eventually experience a VTE.

## Conclusions

Surgical patients at this Central Florida hospital experienced DVT or PE at a rate 0.53% above the national average from 2021 to 2022. Better visibility and adherence to the Caprini risk score tool, in conjunction with routine application of mechanical prophylaxis and early ambulation, may improve outcomes related to VTE at this healthcare facility.

Consistent utilization of DVT and PE risk stratification tools, such as the Caprini risk score calculator, is essential in the prevention of postoperative VTE. Hospitals can improve the utilization of such a tool, thereby reducing the number of embolic events, by making it more visible and accessible to the overseeing provider in the EMR.
